# Zika Virus Infection Presenting with Postauricular Lymphadenopathy

**DOI:** 10.4269/ajtmh.16-0096

**Published:** 2016-08-03

**Authors:** Thomas Weitzel, Claudia P. Cortes

**Affiliations:** ^1^ Programa Medicina del Viajero, Clínica Alemana de Santiago, Facultad de Medicina Clínica Alemana, Universidad del Desarrollo, Santiago, Chile; ^2^ Laboratorio Clínico, Clínica Alemana de Santiago, Facultad de Medicina Clínica Alemana, Universidad del Desarrollo, Santiago, Chile; ^3^ Departamento de Infectología, Clínica Santa María, Santiago, Chile; ^4^ Departamento de Medicina, Escuela de Medicina, Universidad de Chile, Santiago, Chile

**Figure 1.**
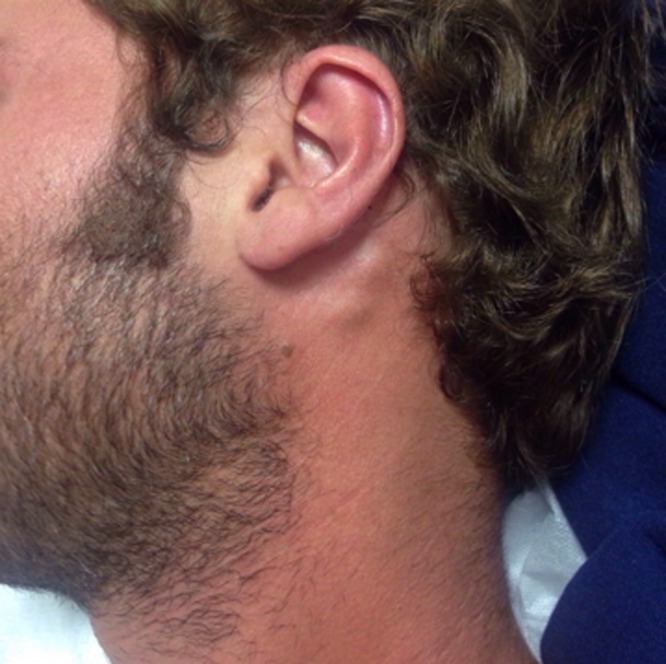
Bilateral tender postauricular lymphadenopathy in patient with Zika virus infection.

**Figure 2.**
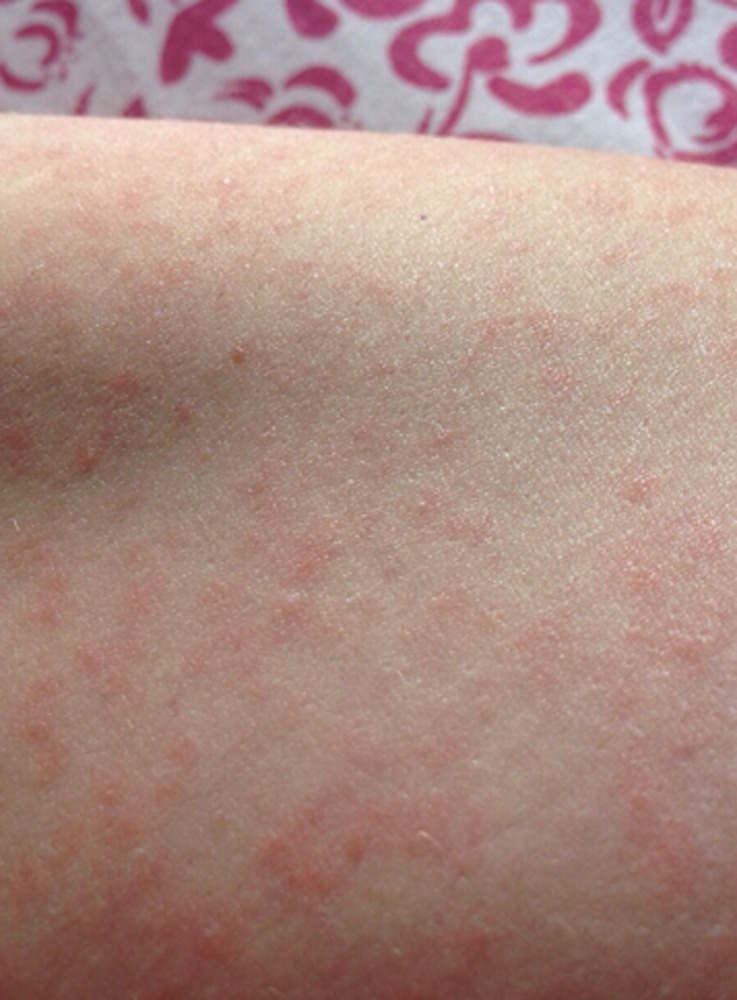
Maculopapular rash on the patient's arm.

A 28-year-old, otherwise healthy Chilean man presented in December 2015 with fever, headache, and myalgia. He had returned, 2 days ago, from a tourist trip to Colombia, where he had visited Bogota and the northern region including Cartagena de Indias, Santa Marta, and Tayrona National Park. During his return, he suffered nonspecific symptoms including sore throat, anorexia, and myalgia. A day later, he noted high grade fever and tender nodules behind his ears (Figure 1

). Physical examination revealed fever (39.0°C), a maculopapular rash of the trunk and extremities (Figure 2

), mild conjunctivitis, and a generalized lymphadenopathy with palpable tender axillary, cervical, and bilateral postauricular lymph nodes. After dengue and chikungunya virus infections were excluded by molecular methods, antigen detection, and IgM antibody testing, samples were sent to the national reference laboratory (Instituto de Salud Pública de Chile, Santiago, Chile), where Zika virus (ZIKV) nucleic acids were detected by real-time reverse transcription polymerase chain reaction (RT-PCR) as previously described.^1^ The sample was also positive using a commercial RT-PCR assay for the detection of ZIKV (Zika Virus genesig^®^ Advanced Kit; Primerdesign^™^ Ltd., Southampton, United Kingdom) in the clinical laboratory, Clínica Alemana, Santiago. The patient recovered rapidly and without complications.

Since 2015, ZIKV is rapidly emerging within the Americas, where it is disseminated by mosquitos of the *Aedes* genus. However, the virus is also transmitted by blood transfusion and, as recently suggested, by contact with infectious semen.^2^,^3^ Because of its possible association with fetal malformations and neurological complications, this epidemic has been declared a global public health emergency by the World Health Organization. The clinical presentation of the infection is similar to dengue including fever, rash, joint pain, conjunctivitis, myalgia, headache, and vomiting.^4^–^6^ Although lymphadenopathy has recently been described in patients with ZIKV infection in Brazil,^7^,^8^ it is usually not listed as a typical manifestation. Our case confirms that ZIKV might cause systemic lymphadenopathy including the posterior auricular lymph nodes. Because tender bilateral postauricular lymphadenopathy is a known clinical sign of postnatal rubella, it might mislead ZIKV diagnosis especially in pediatric patients.
